# Factors affecting subjective cognitive decline in older adults based on the health ecological model: a scoping review

**DOI:** 10.3389/fpsyt.2026.1825454

**Published:** 2026-04-28

**Authors:** Mengfan He, Yulan Chen, Youhua Zhang, Lingmei Ruan

**Affiliations:** 1 School of Nursing, Dali University, Dali, Yunnan, China; 2 Department of Cardiology, The Second People’s Hospital of Kunming, Kunming, Yunnan, China; 3 Anesthesiology Department, Qujing Maternal and Child Health Hospital, Qujing, Yunnan, China

**Keywords:** older adults, subjective cognitive decline, health ecological model, influencing factors, scoping review

## Abstract

**Objective:**

To systematically review and synthesize the factors influencing subjective cognitive decline (SCD) in older adults based on the Health Ecological Model (HEM), and to provide direction for future research.

**Methods:**

Relevant literature on factors influencing SCD in older adults was retrieved from five databases (CNKI, Wanfang, VIP, PubMed, and Web of Science) from database inception to October 18, 2025.

**Results:**

A total of 33 articles were included, comprising 21 Chinese and 12 English articles, with sample sizes ranging from 212 to 74,472 participants. The influencing factors were diverse and complex. According to the HEM, these factors were categorized into four dimensions: personal characteristics, behavioral characteristics, interpersonal networks, and living and working conditions. Notably, none of the 33 studies addressed the fifth dimension—the policy environment—highlighting a significant research gap.

**Conclusion:**

The reported prevalence of SCD varies considerably across assessment tools and geographic locations, particularly in rural areas and among older adults. Four dimensions of influencing factors were identified: personal characteristics, behavioral characteristics, interpersonal networks, and living and working conditions. None of the 33 included studies addressed the policy environment dimension, highlighting a significant research gap. Limitations include the predominant use of self-reported tools, reliance on cross-sectional designs, and geographic concentration, limiting generalizability. Future research should integrate objective methods, prioritize longitudinal designs, investigate policy influences, and include diverse populations for culturally tailored prevention.

## 1 Introduction

Subjective Cognitive Decline (SCD) is an early manifestation of Alzheimer’s Disease (AD) and a precursor to Mild Cognitive Impairment (MCI). It refers to an individual’s self-perceived decline in cognitive function, which may occur despite normal performance on objective tests (1–3). As people age, brain atrophy, reduced neurotransmitter levels, and a decline in cognitive reserve make older adults more susceptible to SCD (4). In China, the prevalence of SCD among the older adults reaches 46.4%, with rates of 38.0%, 45.2%, and 60.3% for those aged 60-69, 70-79, and 80+ years, respectively (5). Studies have shown that the probability of older adults with SCD progressing to MCI or dementia within 10 years exceeds 75%, compared to less than 25% for those without SCD (6). Therefore, SCD represents a critical period for the early prevention of AD, and identifying its influencing factors is crucial for formulating effective prevention strategies.

**Figure 1:**
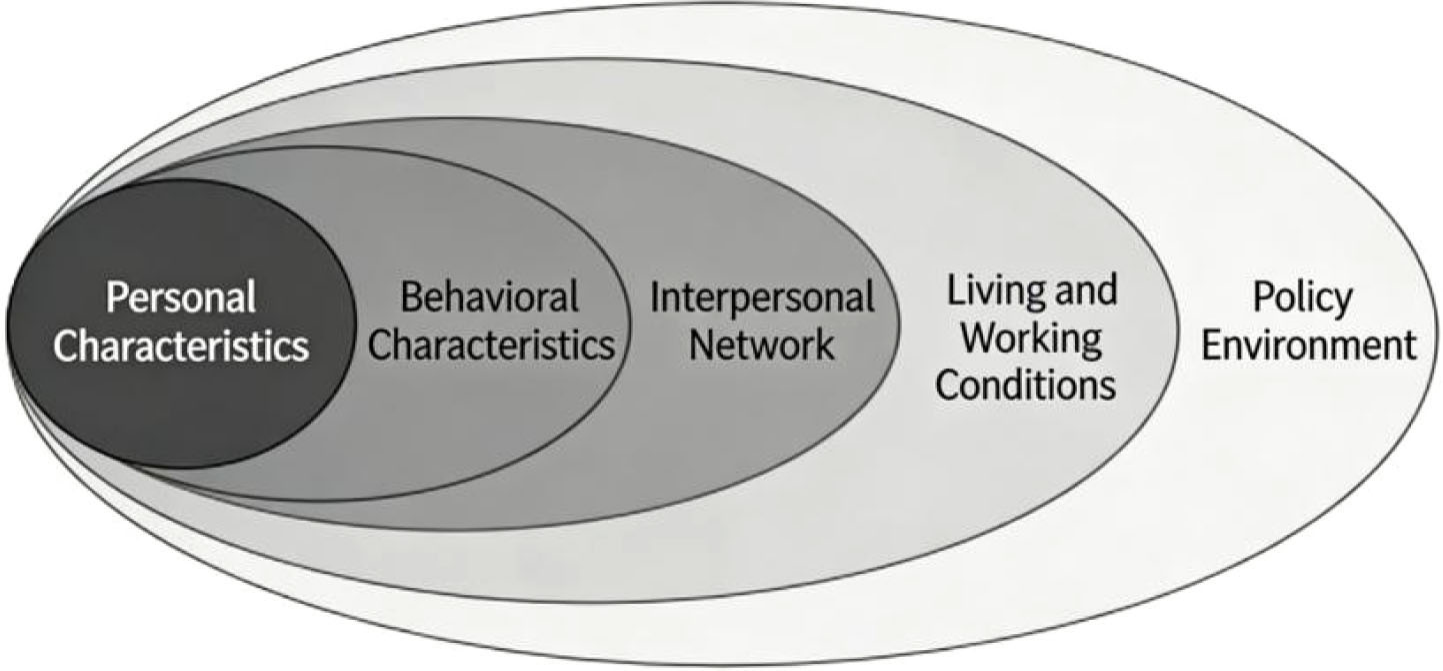
Conceptual framework of the Health Ecological Model (HEM).

The Health Ecological Model(HEM), proposed by Dahlgren and Whitehead in 1991, posits that individual health is the result of interactions among multiple levels of factors, encompassing five dimensions: personal characteristics, behavioral characteristics, interpersonal networks, living and working conditions, and policy environment. This model transcends the limitations of the traditional biomedical model, which often focuses on single factors, and provides a systematic, integrative analytical framework for understanding complex health issues (7). The conceptual framework diagram is shown in **Figure 1**. In studies on the influencing factors of SCD, single-factor analyses often fail to capture the complex, multi-factor interaction mechanisms involved. In contrast, the HEM enables a systematic integration of multiple dimensions—including individual genetics, behavioral habits, social support, living environment, and macro-level policies—thereby facilitating a comprehensive understanding of the occurrence and development of SCD. Therefore, based on this model, the present study systematically reviews the influencing factors of SCD from the perspectives of individual characteristics, behavioral characteristics, interpersonal networks, and living and working conditions, aiming to provide evidence-based references for future research and individualized clinical prevention.

## 2 Materials and methods

This study is based on the methodological framework of a scoping review, and the research question was defined according to the PCC (Population, Concept, Context) framework: P (Population) refers to older adults aged ≥60 years; C (Concept) refers to the influencing factors of SCD; and C (Context) refers to community settings, nursing homes, and general population environments. On this basis, the PRISMA extension for scoping reviews (PRISMA-ScR) developed by Tricco et al. (8) was used as the reporting guideline.

### 2.1 Literature search

Relevant literature on factors influencing SCD in older adults was retrieved from five databases: CNKI, Wanfang, VIP, PubMed, and Web of Science. The search period covered from the inception of each database to October 18, 2025. A combination of subject terms and free-text words was used. Key search terms included: older adults, elderly, advanced age, subjective cognitive decline, risk factors, influencing factors, predictive factors, related factors, and causes. For English databases, taking PubMed as an example, the search strategy was:

#1:(“Aged”[Mesh]) OR ((((((Aged[Title/Abstract]) OR (elder*[Title/Abstract])) OR (old people[Title/Abstract])) OR (older people[Title/Abstract])) OR (older adult[Title/Abstract])) OR older adults[Title/Abstract]))
#2:Subjective Cognitive Decline[Title/Abstract]
#3:((((((((((((((((((Factor, Risk[Title/Abstract]) OR (Risk Factor[Title/Abstract])) OR (Population at Risk[Title/Abstract])) OR (Populations at Risk[Title/Abstract])) OR (Risk Scores[Title/Abstract])) OR (Risk Score[Title/Abstract])) OR (Score, Risk[Title/Abstract])) OR (Risk Factor Scores[Title/Abstract])) OR (Risk Factor Score[Title/Abstract])) OR (Score, Risk Factor[Title/Abstract])) OR (Health Correlates[Title/Abstract])) OR (Correlates, Health[Title/Abstract])) OR (Social Risk Factors[Title/Abstract])) OR (Factor, Social Risk[Title/Abstract])) OR (Factors, Social Risk[Title/Abstract])) OR (Risk Factor, Social[Title/Abstract])) OR (Risk Factors, Social[Title/Abstract])) OR (Social Risk Factor[Title/Abstract])) OR (“Risk Factors”[Mesh])
#4:#1 AND #2 AND #3

Inclusion criteria: (1) Literature published in Chinese or English; (2) Study participants were older adults aged 60 years and above; (3) The research topic was subjective cognitive decline.

Exclusion criteria: (1) Duplicate publications; (2) Reviews, case reports, conference proceedings, or qualitative studies that cannot be quantitatively analyzed for prevalence and influencing factors; (3) Literature where only the abstract was available or the full text could not be obtained; (4) Retracted literature; (5) Studies with missing or incorrect original data; (6) Literature on patients clinically diagnosed with mental illness.

### 2.2 Literature screening and data extraction

**Figure 2:**
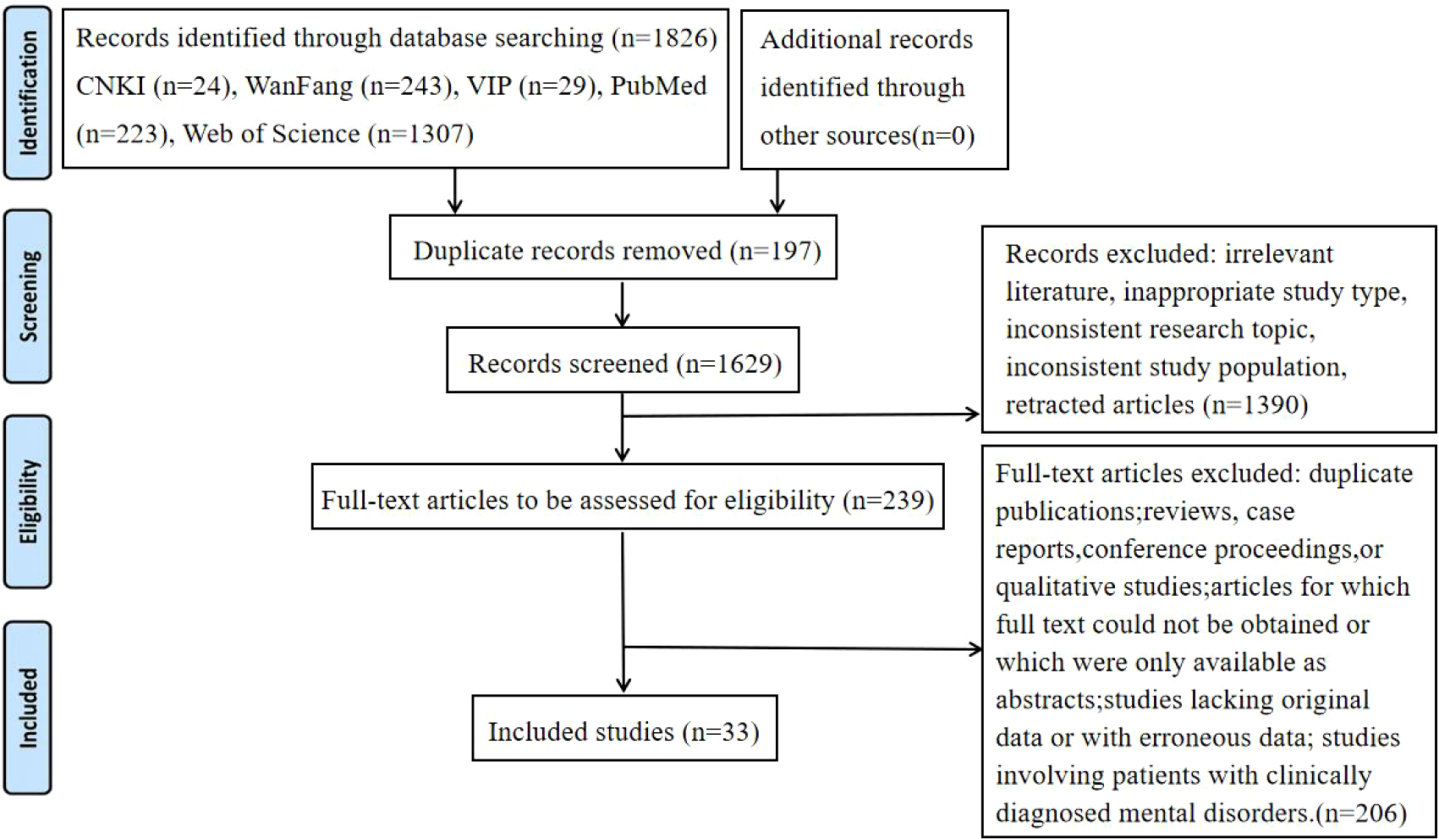
Flow diagram of the literature screening process.

Two researchers independently screened the literature and extracted data, then cross-checked their findings. Any discrepancies were resolved through discussion with a third researcher. Cohen’s Kappa coefficient was calculated using SPSS 26.0 software, yielding a Kappa value of 0.83(95%*CI*:0.62-1.00, *P* = 0.002),indicating substantial agreement between the two reviewers. The screening process was conducted using Zotero reference management software. Titles and abstracts were first reviewed for initial screening, followed by full-text review to determine final inclusion. The literature screening process is illustrated in **Figure 2**. Extracted information included study authors (year of publication), study type, region, sample size, assessment tools, prevalence, and influencing factors.

## 3 Results

### 3.1 Basic characteristics of included studies

A total of 33 articles (1, 9–40) were included, comprising 21 Chinese articles (1, 9–28) and 12 English articles (29–40). Among these, six studies (9–12, 19, 20) investigated SCD among older adults in Anhui Province, four studies (1, 14, 15, 33) focused on Guangzhou, and four studies (22, 30, 34, 35) were nationwide. The remaining 19 studies were conducted in Beijing (n=2) (13, 21), Guangxi (n=1) (16), Zhejiang (n=2) (17, 24), Henan (n=1) (25), Hebei (n=1) (23), Shandong (n=1) (18), Shanghai (n=2) (26, 28), Hubei (n=1) (27), Shenzhen (n=1) (29), as well as other countries including Japan (n=1) (31), South Korea (n=1) (35), the United States (n=1) (36), the United Kingdom (n=1) (38), Malaysia (n=1) (37), Switzerland (n=1) (39), and Germany (n=1) (40). The primary study types were cross-sectional (n=27) (1, 9–15, 17, 19–26, 28, 31–38, 40), followed by cohort studies (n=4) (18, 27, 30, 39), case-control studies (n=1) (29), and mixed-methods studies (n=1) (16). Sample sizes ranged from 212 to 74,472 participants. The prevalence of SCD varied widely, from 3.88% to 83.0%. The basic characteristics of the included studies are summarized in **Table 1**. It should be noted that during the literature screening process, some studies were published by the same research team as both a dissertation and a journal article based on the same dataset. Although all such publications met the inclusion criteria and are therefore presented in **Table 1**, to avoid double-counting of data and to ensure a balanced representation of the evidence, these overlapping entries were treated as a single data source when calculating prevalence rates and influencing factors. Specifically, the following three pairs of studies with overlapping data were analyzed primarily using the journal article versions: Dong Yaqin (2022) (9) and Dong Yaqin et al. (2022) (10); Gu Huaicong (2023) (11) and Gu Huaicong et al. (2023) (12); Zhang Bo (2024) (19) and Zhang Bo et al. (2023) (20).

**Table 1:** Basic characteristics of included studies.

Study author (publication year)	Study type	Region	Sample size	Evaluation tool	Prevalence(%)	Influencing factors
Dong Yaqin (2022) (9);Dong Yaqin et al. (2022) (10)	Cross-sectional	Anhui	264	SCD-Q9	57.95	Low grip strength, female gender, advanced age, insomnia
Gu Huaicong (2023) (11); Gu Huaicong et al. (2023) (12)	Cross-sectional	Anhui	603	SCD-Q9	65.5	Gender, age, smoking, alcohol consumption, education level, household income, HDL, TC/HDL ratio, LDL/HDL ratio
Hao Lixiao et al. (2023) (13)	Cross-sectional	Beijing	2674	SCD-Q9	—	Gender, age, years of education, number of close friends, relationship with neighbors, HAMA score, HAMD score, as well as comorbidities such as cerebrovascular disease, hypertension, hyperlipidemia, and epilepsy
Hu Qing (2022) (14)	Cross-sectional	Guangzhou	414	SCD-Q9	37.2	Advanced age, diabetes, hyperlipidemia, depressive symptoms, anxiety, sleep quality, tea drinking
Hu Qing et al. (2022) (15)	Cross-sectional	Guangzhou	212	SCD-Q9	40.57	Advanced age, anxiety and depressive symptoms, tea drinking
Qin Fan (2025) (16)	Mixed-methods	Guangxi	285	SCD-Q9	61.4	Age, gender, educational level, exercise habits, comorbidity level of chronic diseases, hearing impairment, duration of afternoon nap, ability of daily living activities, anxiety
Wang Jialu et al. (2024) (17)	Cross-sectional	Zhejiang	1004	SCD-Q21	—	Hearing status, visual status, number of medications, Educational level, pain level, apathy status, depression status, hypertension, and whether there is a decrease in grip strength
Xu Shan (2022) (18)	cohort study	Shandong	2488	MMSE、AD8	40.07	Advanced age, illiteracy, APOEϵ4 genotype, low social support, and depressive symptoms
Zhang Bo (2024) (19); Zhang Bo et al. (2023) (20)	Cross-sectional	Anhui	603	SCD-Q9	65.5	Low grip strength, smoking behavior, low education level, advanced age, high waist circumference, high cholesterol
Zhou Ying et al. (2021) (21)	Cross-sectional	Beijing	337	SCD-Q9	32.9	Sleep disorders, anxiety, depression, negative life events, and activities of daily living
Liu Haihong et al. (2023) (22)	Cross-sectional	China	2969	Self-assessment memory tool	—	Physical dysfunction, age, total depression score, self-rated health, education level, total entertainment score, cleanliness, siesta duration, total IADL score, and whether there is broadband
Song Yinhua et al. (2023) (1)	Cross-sectional	Guangzhou	612	SCD-Q9	40.8	Age, education level, sleep quality, anxiety symptoms, poor ADL, and comorbidity of multiple chronic diseases
Wang Bingfei et al. (2024) (23)	Cross-sectional	Hebei	300	SCD-24	61.67	In the past two years have experienced negative life events, cognitive activities
Wang Jialu et al. (2024) (24)	Cross-sectional	Zhejiang	595	SCD-Q21	55.9	Hearing status, visual status, whether there is a decline in grip strength, hypertension, depression, number of medications, physical activity level, and education level
Wu Huimin et al. (2025) (25)	Cross-sectional	Henan	1163	SCD-Q9	83.0	Female gender, self-perceived aging, being unmarried, depression, anxiety, high cognitive function level, and perceived good physical health
Wu Haiping et al. (2024) (26)	Cross-sectional	Shanghai	902	SCD-Q9	21.1	Female, frail, years of education
Huang Yuwei (2022) (27)	cohort study	Hubei	10312	SCD-Q9	48.93	Female, residing in rural areas, unmarried, with low educational level, engaged in manual labor before retirement, suffering from cerebrovascular disease, anemia, and with a history of brain collision and brain trauma
Liu Zheng et al. (2024) (28)	Cross-sectional	Shanghai	1213	SCD-Q9	40.2	Age, education level, number of children, smoking status, daily sleep duration, and depression status
Sun et al. (2025) (29)	Case-control study	Shenzhen	270148	AD8	3.88	Diabetes, anxiety symptoms, depressive symptoms, past smoking history, occasional alcohol consumption, exercise
Cheng et al. (2023) (30)	cohort study	China	10474	MMSE、MoCA	58.33	Married, educated, no living siblings, living alone, lack of close friends, insufficient neighborhood interaction, combined coronary artery disease
Goda et al. (2020) (31)	Cross-sectional	Japan	300	MMSE	35.3	Age, health literacy, depression
Lee et al. (2025) (32)	Cross-sectional	South Korea	74472	Self-assessment memory tool	37.34	Age, subjective health status, subjective stress, and depressive mood
Lin et al. (2022) (33)	Cross-sectional	Guangzhou	688	SCD-Q9	58.4	Urolithiasis, gout, poor sleep quality, depressive symptoms, and anxiety symptoms
Luo et al. (2024) (34)	Cross-sectional	China	428	SCD-Q9	32.2	Subjective health status, lack of physical exercise, visual impairment, and years of education
Ma et al. (2024) (35)	Cross-sectional	China	1120	SCD-Q9	43.8	Obesity, vegetarian diet, long-term smoking, diabetes, coronary heart disease, visual impairment, no spouse, average sleep duration less than 6 hours per night
Pluim et al. (2025) (36)	Cross-sectional	USA	288	ECog	—	Depression, anxiety, and decreased sleep quality
Nazri et al. (2023) (37)	Cross-sectional	Malaysia	293	Self-assessment memory tool	24.6	Having a large waistline, suffering from diabetes, and experiencing depression
Schlosser et al. (2020) (38)	Cross-sectional	UK	491	Neuro-QoL	24.2	Age, gender, education level
Schrempft et al. (2024) (39)	cohort study	Switzerland	1414	SCD-Q9	18.9	Health issues, unhealthy lifestyle habits, depressive symptoms, deteriorating mental health, reduced social activities, and increased feelings of loneliness
Zollinger et al. (2023) (40)	Cross-sectional	Germany	1030	Self-developed assessment tool	31.3	Depression, sleep problems, and higher education level

*SCD-Q, Subjective Cognitive Decline-Questionnaire; MMSE, Mini-Mental State Examination; AD8, Ascertain Dementia 8; HAMA, Hamilton Anxiety Rating Scale; HAMD, Hamilton Depression Rating Scale; HDL, High-Density Lipoprotein; LDL, Low-Density Lipoprotein; TC, Total Cholesterol; BMI, Body Mass Index; APOEϵ4, Apolipoprotein E ϵ4.*.

### 3.2 Influencing factors

#### 3.2.1 Personal characteristics

Demographic Characteristics: Advanced age (1, 9–16, 18–20, 22, 28, 31, 32, 38) and female gender (9, 13, 16, 25–27, 38)were identified as significant risk factors for SCD. In contrast, longer years of formal education (1, 12, 13, 16, 17, 22, 24, 26, 34, 38, 40) and higher educational attainment (28, 30) were associated with a lower likelihood of SCD, compared with being illiterate (18) or having low education levels (19, 20, 27). Older adults without a spouse (25) or those who were unmarried (including single, divorced, or widowed) (27) had a higher incidence of SCD than their married counterparts (30); the number of children (28) was also found to be an influencing factor. Huang (27) suggested that older adults engaged in manual labor before retirement warrant particular attention regarding changes in cognitive function.

Health Status: Older adults with lower self-rated health scores (22), more negative self-perceptions of ageing (25), and a greater number of health problems (39) were at higher risk of cognitive decline. Similarly, Lee et al. (32) and Luo et al. (34) reported that poorer subjective health status was associated with increased SCD risk, indicating that perceived health status may significantly influence actual cognitive health. Among the 33 included studies, 13 indicated that multimorbidity (1, 16), including cerebrovascular disease (13, 27), coronary artery disease (30), coronary heart disease (35), hypertension (13, 17, 24), hyperlipidemia (13), hypercholesterolemia (11, 12, 19, 20), epilepsy (13), diabetes (29, 35, 37), anemia (27), history of cerebral concussion (27), history of traumatic brain injury (27), urolithiasis (33), and gout (33), were independent influencing factors for SCD. As the number of comorbidities increases, the number of medications (17, 24) used also increased. Older adults taking multiple medications were more susceptible to adverse drug effects, which may increase the likelihood of adverse events, poor health outcomes, and cognitive decline (41). A meta-analysis showed that 6% of community-dwelling older adults have both multimorbidity and frailty (26), and 16% of older adults with multimorbidity exhibit frailty, indicating that multimorbidity increases the risk of frailty (42). Studies have suggested that more pronounced frailty is associated with faster cognitive decline (25), and Wu et al. (26) also confirmed a strong correlation between SCD and frailty. Frailty can be assessed by grip strength (43), and several studies have found that grip strength (9, 10, 17, 19, 20, 24) is also associated with SCD. Compared with physically healthy individuals, those with physical dysfunction (22), including hearing impairment (16, 17, 24), visual impairment (17, 24, 34), sleep disorders (1, 9, 10, 14, 21, 33, 36, 40) or average nighttime sleep duration less than 6 hours (28, 35), and mobility limitations (1, 16, 21, 22, 24), are at higher risk for SCD. In addition, mental and psychological problems (39) in older adults, such as anxiety (1, 13–15, 21, 25, 29, 33, 36) and depressive symptoms (13–15, 17, 18, 21, 22, 24, 25, 28, 29, 31–33, 36, 37, 39, 40), are also significantly associated with SCD. When older adults experience depression and anxiety, the likelihood of cognitive decline increases (44). Older adults with higher levels of pain (17) are more prone to apathy (17) and depression compared to those with lower pain levels, which may in turn lead to cognitive decline. Studies by Zhang (19, 20) and Nazri et al. (37) have shown that anthropometric measures, such as waist circumference, are negatively correlated with SCD, and obesity (35) in older adults is also associated with SCD. Regarding genetic factors, the role of the APOEϵ4 allele in memory decline and in the progression from SCD to MCI or to AD over time has been clearly established (45, 46), consistent with Xu’s findings (18). The emergence of health problems also highlights the importance of health literacy. Goda et al. (31) suggested that older adults in communities with low health literacy have a significantly increased incidence of SCD.

#### 3.2.2 Behavioral characteristics

In this study, several unhealthy lifestyle habits (39) were identified as associated with SCD. Seven studies (11, 12, 19, 20, 28, 29, 35) found that smoking was associated with an increased risk of SCD, while three studies (11, 12, 29) suggested that alcohol consumption was associated with an increased risk of SCD. Tea drinking (14, 15) and long-term vegetarianism (35) may also affect the occurrence of SCD, and failing to develop good napping habits or experiencing poor napping quality (16, 22) can also promote the occurrence of SCD. Older adults who engage in regular exercise (16, 29) have a lower probability of developing SCD, whereas a lack of physical activity (34) may accelerate cognitive decline. Liu et al. (22) showed that participation in recreational activities such as playing chess, cards, dancing, and cognitive training exercises is beneficial for maintaining cognitive function (23). Additionally, the cleanliness of one’s living environment (22) can positively predict cognitive function in older adults with SCD.

#### 3.2.3 Interpersonal network

The study by Hao et al. (13) indicated that older adults with more close friends and better relationships with neighbors had lower SCD scores. Conversely, factors such as a lack of close friends (30), having no surviving siblings (30), low levels of social participation (39), or insufficient neighborhood interactions (30), along with the resulting increase in loneliness (39), were all associated with a higher risk of SCD. These findings also highlight the importance of family and social support (18) in addressing SCD.

#### 3.2.4 Living and working conditions

This study also revealed a negative correlation between household income (11, 12) and SCD. Older adults living alone (30) were at a higher risk of developing SCD. Furthermore, the overall prevalence of SCD was higher among older adults living in rural areas (55.9%) than among those in urban areas (39.2%) (27), which may be attributed to differences in economic development, cultural background, and the availability of medical and health resources. For older adults living in urban areas, having broadband internet access at home (22) facilitates exposure to online resources, which has been shown in nursing studies conducted in countries such as Sweden to have a positive impact on cognitive function (47). In addition, older adults who experienced more negative life events in recent years (21, 23) exhibited accelerated cognitive decline, which is associated with changes in mood, psychological state, and personality.

## 4 Discussion

The prevalence of SCD varies considerably across studies (3.88%–83.0%), a disparity likely attributable to differences in assessment tools, geographical regions, and the age composition of study populations, with higher rates consistently reported in rural areas and among older adults. This study identified the Subjective Cognitive Decline Questionnaire (SCD-Q), the Mini-Mental State Examination (MMSE), and the Memory Impairment Self-Rating Scale (AD8) as the most commonly used assessment tools. It is important to note that while the MMSE is an objective cognitive screening tool and conceptually differs from the core definition of SCD (i.e., self-perceived decline despite normal objective performance), several included studies employed it to identify or screen for SCD. In these instances, its primary purpose was to exclude individuals with objective cognitive impairment during population screening, thereby ensuring participants met the prerequisite of “normal objective cognition.” Consequently, these studies were included not because the MMSE was used to define SCD directly, but because it served as a screening mechanism to establish the required cognitive baseline. However, the reliance on such objective tools may introduce conceptual heterogeneity, as studies that use only objective measures may not fully capture the subjective experience central to SCD. This variation in assessment approaches may influence both the reported prevalence of SCD and the interpretation of its associated factors, potentially leading to overestimation or underestimation of certain associations. When applying these tools, context is crucial: the MMSE is well-suited for large-scale community screening, whereas the SCD-Q is more appropriate for in-depth assessment of subjective experiences. Furthermore, adjustments should be made for local cultural characteristics to avoid assessment bias.

Regarding personal characteristics, advanced age and female gender are non-modifiable risk factors. Their association with SCD is likely mediated by age-related brain atrophy and the diminished neuroprotective effects of estrogen after menopause, underscoring the need for regular health monitoring for early identification. Conversely, low educational attainment (which reduces cognitive reserve) and chronic disease comorbidity (which affects cerebral blood flow) are potentially modifiable. Interventions such as cognitive training (e.g., memory skills training, logic games) can help compensate for reduced cognitive reserve, and these should be combined with effective disease management and cognitive monitoring for a dual-intervention approach.

For behavioral characteristics, unhealthy habits such as smoking and physical inactivity were found to increase SCD risk. Smoking impairs cerebral vascular elasticity, leading to insufficient cerebral perfusion, while a lack of exercise reduces cerebral blood flow and neurotransmitter secretion. Interventions require tailored health education that considers the physical tolerance of older adults. Highlighting the benefits of moderate napping, which can compensate for insufficient sleep and promote neural repair, is a low-cost and scalable intervention that can be promoted through community guidance.

Within interpersonal networks, a lack of close friends and insufficient neighborhood interaction are related to SCD. Communities can build social platforms and organize activities such as chess, dance, and crafts to achieve the dual benefits of social interaction and cognitive engagement. Such activities require logic, memory, and coordination, which can alleviate loneliness and stimulate cognitive function, especially among empty-nest and solitary older adults.

In the dimension of living and working conditions, the prevalence of SCD in rural areas is significantly higher than in urban areas, mainly due to insufficient medical resources and low coverage of chronic disease control and cognitive screening. Solitary older adults are prone to having early cognitive decline signals overlooked due to a lack of caregivers. Strategies to mitigate this could include strengthening grassroots cognitive health services to improve access to resources and providing regular on-site assessments for solitary older adults, thereby integrating cognitive monitoring into overall health management.

In summary, interventions across dimensions do not exist in isolation but rather form a mutually reinforcing synergy. Chronic disease screening at the individual characteristic dimension depends on the implementation of primary healthcare resources within the living and working conditions dimension. Regular exercise at the behavioral characteristic dimension can be supported by group supervision through community activities within the interpersonal network dimension, thereby improving adherence. The establishment of social platforms within the interpersonal network dimension can alleviate symptoms such as anxiety and depression at the individual characteristic dimension, reducing the detrimental effects of negative emotions on cognition. This multi-dimensional, synergistic approach can maximize SCD risk reduction and provide a foundation for developing individualized and systematic prevention strategies in clinical practice. Furthermore, none of the 33 included studies addressed the fifth dimension of the HEM—the policy environment—a gap that in itself constitutes an important finding. The absence of policy environment factors in SCD research may reflect that current studies remain largely focused on the individual level, without sufficiently attending to the mechanisms through which macro-level policies influence cognitive health. This may be attributed to several factors, including the relatively recent introduction of the SCD concept, insufficient interdisciplinary collaboration in policy-oriented research, and the inherent challenges in evaluating the effectiveness of policy interventions. In terms of existing older adults care policies, although China has introduced policy documents such as the *Healthy China Initiative (2019-2030)*, which explicitly call for strengthening cognitive screening and intervention among older adults, the implementation of these policies at the grassroots level still faces challenges, including a lack of unified implementation standards, insufficient resource allocation, and limited health insurance coverage. For instance, community-based cognitive screening has not yet been incorporated as a routine component of basic public health services in some regions, and cognitive interventions such as cognitive training and psychological support are not covered by health insurance. These limitations have, to some extent, hindered the accessibility and sustainability of early SCD identification and prevention efforts. This research gap suggests that future studies should strengthen theoretical development and empirical investigation into the relationship between the policy environment and SCD, and promote the integration of cognitive health in older adults into public health policy frameworks. Such efforts may include implementing community-based cognitive screening services, conducting public education initiatives on cognitive health, and expanding health insurance coverage for cognitive intervention programs, thereby providing support for the early identification and comprehensive management of SCD at the policy level. Finally, among the 33 studies included in this review, 26 were conducted in China. This geographic concentration may limit the generalizability of the findings, as differences in healthcare systems, lifestyle patterns, social support structures, and cultural attitudes toward cognitive decline may influence the prevalence and risk factors of subjective cognitive decline in ways that differ from those observed in Chinese populations. Therefore, future research should include more diverse populations to better understand how variations in culture, the economy, and healthcare systems shape the risk factors and manifestations of subjective cognitive decline.

## 5 Limitations and future directions

This study has several limitations: (1) The included studies predominantly relied on self-reported assessment tools, which may introduce recall and reporting biases. Future research should combine subjective measures with objective detection methods, such as neuroimaging and blood biomarkers, to enhance the validity and objectivity of the findings. (2) The majority of the included studies were cross-sectional in design, which can only identify associations and cannot establish causal relationships. Longitudinal cohort or case-control studies are needed to clarify the temporal sequence and causal pathways between influencing factors and subjective cognitive decline. (3) None of the 33 studies addressed the policy environment dimension of the HEM, indicating a significant gap in understanding how macro-level policies influence cognitive health. Future research should examine the impact of policy documents on the accessibility and quality of cognitive health services. (4)The geographic concentration of the included studies limits the generalizability of the findings. Future research should include more diverse populations and provide a basis for developing culturally tailored prevention strategies.(5)Regarding the assessment of SCD, the inclusion of studies using objective screening tools such as the MMSE may introduce conceptual heterogeneity and may not fully capture the subjective experience central to SCD. This variation in assessment approaches may affect the reported prevalence and influencing factors of SCD, potentially leading to overestimation or underestimation of certain associations. Therefore, future research should adopt a standardized assessment framework that clearly distinguishes between objective screening and subjective experience and combines both approaches to provide a more comprehensive understanding of SCD.
